# Time-Delayed Anticancer Effect of an Extremely Low Frequency Alternating Magnetic Field and Multimodal Protein–Tannin–Mitoxantrone Carriers with Brillouin Microspectroscopy Visualization In Vitro

**DOI:** 10.3390/biomedicines12020443

**Published:** 2024-02-16

**Authors:** Anatolii A. Abalymov, Roman A. Anisimov, Polina A. Demina, Veronika A. Kildisheva, Alexandra E. Kalinova, Alexey A. Serdobintsev, Nadezhda G. Novikova, Dmitry B. Petrenko, Alexandr V. Sadovnikov, Denis V. Voronin, Maria V. Lomova

**Affiliations:** 1 Science Medical Centre, Saratov State University, 83 Astrakhanskayast, Saratov 410012, Russia; 2 Institute of Chemistry, Saratov State University, 83 Astrakhanskayast, Saratov 410012, Russia; 3 Institute of Physics, Saratov State University, 83 Astrakhanskayast, Saratov 410012, Russia; 4 Institute of Comprehensive Exploitation, Mineral Resources Russian Academy of Sciences, Moscow 111020, Russia; 5 The Core Shared Research Facility “Industrial Biotechnologies”, Aleksei Nikolayevich Bach Institute of Biochemistry, Russian Academy of Sciences, Moscow 119071, Russia; 6 Geological Institute, Russian Academy of Sciences, Moscow 119017, Russia; 7 Faculty of Natural Sciences, Department of Theoretical and Applied Chemistry, Federal State University of Education, Mytischi 141014, Russia; 8 Department of Physical and Colloid Chemistry, National University of Oil and Gas “Gubkin University”, Moscow 119991, Russia

**Keywords:** magnetic nanoparticles, mitoxantrone, alternative magnetic field, calcium carbonate, breast cancer

## Abstract

The effect of an extremely low frequency alternating magnetic field (ELF AMF) at frequencies of 17, 48, and 95 Hz at 100 mT on free and internalized 4T1 breast cancer cell submicron magnetic mineral carriers with an anticancer drug, mitoxantrone, was shown. The alternating magnetic field (100 mT; 17, 48, 95 Hz; time of treatment—10.5 min with a 30 s delay) does not lead to the significant destruction of carrier shells and release of mitoxantrone or bovine serum albumin from them according to the data of spectrophotometry, or the heating of carriers in the process of exposure to magnetic fields. The most optimal set of factors that would lead to the suppression of proliferation and survival of cells with anticancer drug carriers on the third day (in comparison with the control and first day) is exposure to an alternating magnetic field of 100 mT in a pulsed mode with a frequency of 95 Hz. The presence of magnetic nanocarriers in cell lines was carried out by a direct label-free method, space-resolved Brillouin light scattering (BLS) spectrometry, which was realized for the first time. The analysis of the series of integrated BLS spectra showed an increase in the magnetic phase in cells with a growth in the number of particles per cell (from 10 to 100) after their internalization. The safety of magnetic carriers in the release of their constituent ions has been evaluated using atomic absorption spectrometry.

## 1. Introduction

Responsive biomimetic platforms (either molecular, mineral, or polymeric) are currently considered onward systems for site-specific diagnosis and therapy of socially significant diseases [1,2]. The major benefits of these platforms are the possibility of the targeted and remote-navigated delivery of expensive and/or hazardous drugs, which greatly reduces the required dose and side effects, and there is a possibility to control the drug release at the delivery site [3,4,5]. From a diagnostic perspective, magnetic platforms appear to be the systems for functional imaging using non-invasive label-free Biobrillouin technologies [6].

Magnetically responsive platforms attract much attention owing to their capability to be navigated and induce a drug release by a magnetic field [7]. Magnetic fields have a good penetration ability into biotissues, are non-ionizing, and are considered to be safe for humans within certain limits. Thus, magnetic fields are widely employed in modern medicine practice for diagnosis and therapy, as examined by MRI and magnetic hyperthermia [8,9,10,11,12,13]. To date, it has been demonstrated that a non-uniform static magnetic field is effective for the remote navigation of magnetically responsive drug delivery carriers, including experiments in vivo [14]. On the other hand, drug release can be triggered by an alternating magnetic field (AMF) without a significant increase in the ambient temperature, which significantly increases the perspective of this type of field in therapy [15,16]. Two principal mechanisms are possible depending on the frequency of the applied field. The first one is magnetic hyperthermia, induced by high-frequency AMFs (≥100 kHz). This can be employed for the disruption of thermo-responsive drug carriers localized, for instance, in the tumor site. However, according to some estimations, in biotissue, the thermal field generated by AFM exposure is not spatially localized specifically into or nearby the carriers and may induce the necrosis of the surrounding healthy cells [17]. Alternatively, the exposure to low frequency (100–700 Hz) or extremely low frequency (≤100 Hz) alternating magnetic fields (ELF AMFs) induces the reorientation of the magnetic moment of single-domain superparamagnetic nanoparticles that result in whole particle motion following AMF oscillations [18]. In case the nanoparticles are incorporated into a carrier shell or conjugated to drug-carrying macromolecules, the drug can be released through the magneto-mechanical actuation of the shell rapture or the initiation of direct drug desorption from the carrier surface [17]. Magnetic carriers in LFs and ELF AMFs move around the axis of action of magnetic forces [19], like a vortex. Unlike magnetic hyperthermia, the mechanical actuation induced by non-heating AMFs can be spatially localized specifically to magnetic drug carriers. Additionally, exposure to an alternating magnet results in the improvement of cell wall permeability due to the magnetoporation effect (the process of pores in cells formation under the magnetic field treatment) [20,21,22]. These suggest exposure to ELF AMFs as a promising approach to the site-specific drug release triggered by magnetic fields.

Calcium carbonate in vaterite polymorph modification is a versatile substrate fordesign drug delivery platforms owing to its spherical shape and porous structure. Additionally, a number of studies demonstrate the low toxicity of calcium carbonate particles upon intravascular [23], transdermal [24,25], and oral administration [26], and mucosal delivery [27,28,29]. Therefore, the vaterite particles may be employed as drug carriers themselves by loading therapeutic agents to their pores to either act as a core or a sacrificial template for drug carrier assembly.

In our previous work, we have described the anticancer hybrid drug delivery carriers with mineral vaterite/magnetite cores and protein–tannin shells loaded with doxorubicin [30]. The carriers demonstrated remarkable guidance by the applied magnetic field and good anti-breast cancer activity, which, however, was attended by the spontaneous release of doxorubicin. In this study, we propose anti-breast cancer drug delivery carriers based on the vaterite/magnetite core and protein–tannin–mitoxantrone shell instead. Mitoxantrone is one of the most common anti-breast cancer drugs with a unique mechanism of intracellular metabolism [31], yet it is unstable in the acidic medium intrinsic to tumors [32]. However, according to some reports, this can be avoided by the formation of a highly stable protein–tannin–mitoxantrone complex, which is formed by hydrophilic interactions [33,34,35]. Furthermore, the overall stability of the shell is of great importance, as uncontrolled drug release may lead to side effects in the organism, while the protein release may cause an allergic response.

The study of the magnetic properties of cell lines and their organelles containing magnetic carriers is studied directly in very rare cases. Moreover, in these studies, the researchers are mostly focused on the possibility of the magnetic attraction of biological objects by a permanent magnet, thereby proving the acquisition of magnetic properties by cell lines or biological objects [36]. This approach is indirect, and the selection of a permanent magnet should be approached very thoughtfully, taking into account the magnetic properties of the carriers [37]. With this respect, Brillouin microscopy appears as a versatile and non-destructive technique for the spatially resolved studies of biological objects and/or carriers. The development of Biobrillouin technologies (BioBLS) is now taking place in several major world scientific groups [38,39,40]. In this case, magnetic characterization is not the main one and strongly depends on the types of lasers and recording systems used. Phonon interaction can provide valuable information about the spatial rigidity of biological objects with high resolution, which cannot be obtained by any other method, and the method remains label-free and non-invasive [41]. The multifactor nature of studies using Brillouin spectroscopy puts this method on par with the most popular methods for characterizing biological objects, including fluorescent, photoacoustic, and ultrasonic techniques [42,43].

In this way, this work aims for several goals, namely, to obtain a model object that does not degrade significantly (prove a measure of degradation) under the action of a magnetic field to study the effect of ELF AMFs on 4T1 mouse breast cancer cells in vitro in their presence; to visualize magnetic carriers in cells using label-free non-destructive BioBLS technologies in vitro as an analog of fluorescence methods; and to select parameters of an alternating magnetic field that would inhibit the growth of cancer cells in vitro, taking into account the assessment of cancer cell survival.

## 2. Materials and Methods

### 2.1. Materials

Calcium chloride dihydrate, sodium carbonate (anhydrous), iron(III) chloride hexahydrateiron(II), chloride tetrahydrate, sodium hydroxide, tannic acid (TA), bovine serum albumin (BSA, lyophilized powder) and TRITC-conjugated BSA, phosphate-buffered saline (PBS), citric acid, glycyl alcohol, ethanol, mitoxantrone (Mit), Dulbecco’s modified Eagle’s medium (DMEM) (high glucose), Bradford reagent, phosphate buffer solution (PBS), citric acid, glycyl alcohol, trypsin (from porcine pancreas, ~1500 U/mg), pepsin (from porcine gastric mucosa, ≥2400 U/mg), tris(hydroxymethyl)aminomethane (≥99.8%), and sodium citrate were purchased from SigmaAldrich (Germany, Taufkirchen). Fetal bovine serum (FBS) was purchased from Gibco (Waltham, MA, USA). AlamarBlue was purchased from Thermo Fisher Scientific (Waltham, MA, USA).

Millipore Milli-Q deionized (DI) water (18.2 MΩ·cm) was used in all sets of experiments.

### 2.2. Preparation of Mit-Loaded Magnetic Carriers

**Scheme 1:**
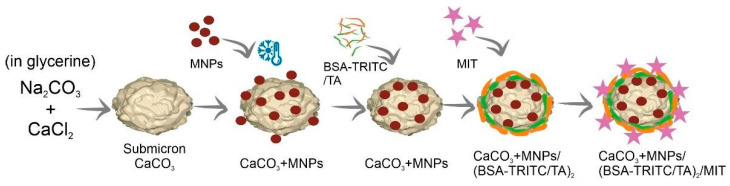
Stepwise preparation of Mit-loaded magnetic carriers.

Mit-loaded magnetic carriers were prepared according to the modified procedure described in reference [30]. The stepwise preparation is presented in Scheme 1. In brief, magnetite Fe_3_O_4_ nanoparticles (MNPs) were prepared according to Massart’s ferrous–ferric salts co-precipitation method [44] with a homemade automated reactor setup [45]. The resulting MNP colloid had a concentration of 2.4 ± 0.1 mg/mL and an average particle size of 11 ± 3 nm. CaCO_3_ particles with the vaterite structure were synthesized according to [46].

Submicron CaCO_3_ particles were loaded with MNPs with the freezing-induced loading (FIL) technique [47]. To perform this, 2 mL of MNP colloid was added to 10 mg of CaCO_3_ particles. The resulting mixture was placed in a freezer at −20 °C in a continuously rotating flask for 2 h to ensure that the aqueous phase was completely frozen. Upon freezing, the CaCO_3_/MNPs particles were thawed at room temperature and rinsed with DI water. Then, the samples were dried at +60 °C or were sent to a repeated freezing/thawing procedure (a loading cycle).

After 1 or 3 loading cycles of MNPs, a protein–tannin shell was layer-by-layer assembled onto the surface of CaCO_3_/MNPs cores. For this purpose, aqueous solutions of BSA (or BSA-TRITC) with a concentration of 3.3 mg/mL and TA with a concentration of 1 mg/mL were iteratively added to CaCO_3_/MNPs particles. As a result, CaCO_3_/MNP_1(3)_/(BSA/TA)_2_ core–shell structures with two BSA/TA bilayers were obtained. Finally, 200 μg of mitoxantrone (Mit) aqueous solution was added to the core–shell structures to conjugate Mit to magnetic carriers. The surface morphology and size distribution of the CaCO_3_ particles and Mit-loaded magnetic carriers were analyzed with a MIRA II LMU scanning electron microscope (Tescan, Brno, Czech Republic) equipped with an INCA Energy 350 energy-dispersive microanalysis system. SEM images were captured at an operating voltage of 30 kV in secondary electron emission mode. The CaCO_3_ particle size distribution was calculated by post-processing and image analysis of the SEM micrographs with ImageJ.net 1.53e software. At least 100 measurements per sample were performed.

### 2.3. Scanning Electron Microscopy (SEM)

The surface morphology and size distribution of the CaCO_3_ particles and Mit-loaded magnetic carriers were analyzed with a MIRA II LMU scanning electron microscope (Tescan, Brno, Czech Republic) equipped with an INCA Energy 350 energy-dispersive microanalysis system. SEM images were captured at an operating voltage of 30 kV in secondary electron emission mode. The CaCO_3_ particle size distribution was calculated by post-processing and image analysis of the SEM micrographs with ImageJ.net 1.53e software. At least 100 measurements per sample were performed.

### 2.4. The Magnetic Hysteresis Loop Measurements

The magnetic hysteresis loops of the CaCO_3_/MNPs particle powder were measured with a PPMS vibrating sample magnetometer (Quantum Design PPMS w/VSM option) at 300 K with an in-plane applied magnetic field up to 9T.

### 2.5. Determination of Protein and Mitoxantrone Content in the Carrier Shell

The protein content in the carrier shell was determined spectroscopically. For this purpose, TRITC-conjugated BSA was used for shell assembly. Upon the deposition of every TRITC-BSA layer, the supernatant was collected, and the residual concentration of TRITC-BSA was calculated by measuring the absorbance at 557 nm (BioTek Synergy H1, Santa Clara, CA, USA).

The mitoxantrone content in carriers was calculated by measuring the adsorbance of supernatants collected after conjugation at 610 nm (BioTek Synergy H1, Santa Clara, CA, USA). The calculation of the cytostatic mass was carried out according to the standard method using a calibration curve.

### 2.6. Extremely Low Frequency Alternating Magnetic Field (ELF AMF) Exposure

The study of ELF AMF exposure to the magnetic carriers was carried out with a TOR 04/17 Combo setup (Nanomaterials, Tambov, Russia). The setup is capable of generating a low frequency magnetic field in the range from 20 to 210 Hz and tunable magnetic field induction up to 130 mT. The setup is equipped with a water-cooling circuit, which allows for the cooling of the elements and the electromagnet assembly by means of a chiller (SMC, Japan, Tokyo). The setup is designed to expose the whole 96-well plate volume, and, thus, generate highly homogeneous ELF AMFs in the working area with the maximal decay of the induction of 5% at the edges. Additionally, the setup is equipped with an IR temperature sensor to monitor the temperature change in the working area during the exposure. All experiments were carried out at a room temperature of +20 °C, which was maintained by a climate control system.

To study the protein release under ELF AMF exposure, the magnetic core–shells CaCO_3_/MNP_1(3)_/(TRITC-BSA/TA)_2_ were dispersed in 0.1 M PBS (sample volume 200 µL) and dropped into the wells of 96-cell plates. The mass of particles per well varied depending on the experimental conditions and is described separately. After that, the cell plates were treated by ELF AMFs. In experiments that require temperature measurements in each well, as well as cell experiments, the samples in the 96-well plate were buried, excluding the outermost perimeter wells, based on the conditions of cell cultivation. For all other experiments, all cells of the 96-well plate were used because the generated low frequency alternating magnetic field has a high degree of homogeneity, as described above. The magnetic field induction was 100 mT, and the frequencies were 95, 48, and 17 Hz. The exposure to the ELF AMF was performed in an operation mode with 5 min of exposure followed by a 30 s break, and again 5 min of exposure. After AMF exposure, the core–shells were sedimented by a permanent magnet. A total of 5 μL of supernatant was collected from each well and added to 250 μL of Bradford reagent. The mixture was kept for 30 min at room temperature, and the absorbance of the protein–dye complex was measured at 595 nm. All absorbance spectra were obtained using an ultraviolet–visible spectrometer CLARIOstar Plus microplate reader (BMG LABTECH GmbH, Ortenberg, Germany).

Analogously, the release of mitoxantrone from Mit-loaded magnetic carriers CaCO_3_/MNP_1(3)_/(BSA/TA)_2_Mit after ELF AMF exposure was measured. After AMF exposure, 120 μL of the supernatant was collected from each well, and the adsorption spectra were measured in a wavelength range of 400–850 nm to determine Mit presence.

In all sets of experiments, the temperature of magnetic carrier suspension before and after ELF AMF exposure was controlled with an InfratecVarioCAM HD head 880 infrared camera (InfraTec, Dresden, Germany). The collected data were processed with standard data processing IRBIS 3.1 plus software and were presented as dependences of the relative temperature change (Δ*T*/*T*) on the treatment condition as follows:

(1) $$\frac{\Delta T}{T}=\frac{T_{after}-T}{T}\times 100\;\%$$

where T is the temperature measured before ELF AMF treatment and $T_{after}$ is the temperature measured after ELF AMF treatment.

### 2.7. 4T1 Cell Culture Cultivation

Mammary gland cancer cells (4T1 cell culture) were cultured in RPMI supplemented with 10% FBS and 1% of penicillin/streptomycin. The media were replaced every 3 days, and the cells were maintained in a humidified incubator at 5% CO_2_ and +37 °C (Innova CO-170, New Brunswick Scientific, Hamburg, Germany).

### 2.8. Cell Viability

Cell viability was measured at different times using a Cell Proliferation Reagent WST-1 on 4T1 cells incubated with carriers loaded by cytostatic with and without ELF AMF treatment. As a control, cells that did not receive cytostatic and ELF AMF treatment were used. 4T1 culture cells were seeded into 96-well plates at a cell density of 10^4^ cells per well. After a day, particles containing 1, 5, and 10 µg/mL of the mitoxantrone and CaCO_3_/MNP_3_/(BSA/TA)_2_Mit were added to the cells. Then, after 1 and 3 days of co-incubation, the cells were exposed to an alternating magnetic field of 95 Hz and 100 mT for 10 min. The cells were then incubated for 4 h, with 10 μL of fluorescent dye added to each well (Cell Proliferation Reagent WST-1, Roche, Basel, Switzerland). At the last step, absorbance at 440 nm was measured using a spectrophotometer (BioTek Synergy H1, Santa Clara, CA, USA). Calcein AM (Ex. 494 nm/Em. 517 nm) and propidium iodide (Ex.535 nm/617 nm) dyes at concentrations of 1 μM and 1 μg/mL were used to stain live/dead cells, respectively. The dyes were added to a Petri dish and incubated with cells for 30 min. Live and dead cells were visualized using an Olympus BX51 fluorescence microscope (Olympus, Tokyo, Japan).

### 2.9. Fluorescent Microscopy

The uptake of carriers inside the cells was examined by growing 50,000 cells for 24 h in 35mm thin-bottomed sterile Petri dishes (Grid-500, ibidi USA, Inc., Fitchburg, WI, USA). After growth for 24 h, 100 μL (10 mg/mL) of an aqueous suspension of particles was added to the Petri dish surface with a concentration of 50 particles/cell, and the samples were incubated for another 24 h. Before confocal laser scanning microscopy (CLSM) measurements, the cell culture medium was removed from the scaffold dishes to discard unabsorbed particles, after which the dishes were washed several times in phosphate-buffered saline (PBS). Cells were fixated by −4 °C ethanol for 15 min. After that, the fixated cells were treated with Triton X-100 for 15 min. To acquire a fluorescence signal from the cells, the cellular environment was stained with Alexa Fluor 488 (Thermo Fisher Scientific, Waltham, MA, USA). Staining was performed as follows: 1 μL of a solution of Alexa Fluor 488 (5 mM) was injected into a scaffold dish containing 1mL of the cell culture. The dish was kept at 37 °C for 45 min in an incubator supplied with a 5% CO_2_ atmosphere. Then, the sample was rinsed once with PBS to remove the unbounded dye. For CLSM, the cell culture was replaced by PBS and was imaged with ax40/NA0.60 objective in both transmission and fluorescent modes using a Leica (Wetzlar, Germany) TCS SP8 X inverted confocal microscope.

### 2.10. Atomic Absorption Spectrometry

Concentrations of Fe and Ca in supernatants were measured by atomic absorption spectrometry (AAS) in an acetylene–air flame on a KVANT-2MT spectrometer (KORTEK, Russia, Moscow). Concentrations of sodium and potassium were determined on a FPA2-01 flame photometer (ZOMZ, SergievPosad, Russia) in a natural gas–air flame. LaCl_3_ and CsCl were used as ionized buffers to determine Ca; their concentrations in the analyzed solutions were 0.1% and 0.01%, respectively. The detection limits of Fe were 5–40 µg/L, and Ca was 100 µg/L. The relative errors of measurement of element concentrations in the solutions did not exceed ±4%. Supernatants were prepared by mixing magnetic calcium carbonate particles in enzymes and buffer solutions following centrifugation, according to the procedure described in [48]. The following samples were characterized by AAS: supernatants from calcium carbonate magnetic particles with protein–tannin shells after incubation in (1) trypsin solution for 0, 15, 30, 45, and 60 min, (2) Tris-HCl buffer, (3) pepsin, and (4) citrate buffer for a similar time. All 20 sample groups had at least 3 replicates.

### 2.11. Brillouin Light Scattering Investigation (2DMapping and a Common BLS Signal) of Magnetite Submicron Carriers, Internalized into Cells

The development of label-free technologies for the visualization of magnetic carriers can be based on the effect of inelastic light scattering, which allows not only to fix the localization of particles but also to characterize their magnetic properties. When a focused optical beam is incident on a substance, a number of effects occur, namely, absorption and scattering of light, which can be used to investigate the properties of the material. Three types of scattering are important here—elastic Rayleigh scattering, inelastic Brillouin scattering, and Raman scattering. The Mandelstam–Brillouin spectroscopy method within the class of analytical methods finds wide application for the analysis of mechanical and viscous properties in soft matter physics, materials science (mainly in homogeneous and equilibrium systems), and solid state physics studies [49,50,51]. At the moment, there are only magnetic and atomic force microscopies, which work in semi-contact or contact modes, which are difficult to realize for cellular systems in vitro and even more difficult to model for vital systems.

To study the Brillouin light scattering (BLS) mapping from CaCO_3_/MNPs_3_/(BSA/TA)_2_particles inside the cells, the population of 50,000 mammary gland cancer cells (4T1 cell culture) was grown by incubation for 24 h in 35mm thin-bottomed sterile Petri dishes (Grid-500, ibidi). After incubation for 24 h, water suspension of CaCO_3_/MNPs_3_/(BSA/TA)_2_particles was dropped in the Petri dishes, maintaining a concentration of 10, 50, and 100 particles/cell, and the samples were incubated for another 24 h. Before BLS measurements, the cell culture medium was removed from the scaffold dishes to discard unabsorbed particles, and the dishes were washed several times with phosphate-buffered saline (PBS).

Assessing the internalization of practically drug carriers into cell lines is fairly easy using fluorescence microscopy if the carriers themselves are ≥1 µm in size. If the carriers are slightly smaller than this size, then estimates are also made, but this is rather difficult [52]. In any case, this method of determining the concentration of carriers in a cell after their introduction is indirect. Another technique for detecting carriers in a cell is 2D/3D mapping using BLS [41]. In this case, magnetic media must have a magnetic label. This method of visualization carriers in a cell is non-invasive, unlike MFM, which, although it can work in a semi-contact mode with cell lines, the quality of the resulting images is not high. In contrast to the fluorescence method, BLS microscopy for cells also makes it possible to construct an averaged BLS spectrum, which provides semi-quantitative information on the content or change in the content of magnetic nanoparticles in cells. Combining the magnetic characteristics of the carriers obtained by SQUID and VSM, and mapping, the averaged integral signal of BLS normalized to the mass of the samples will allow us to proceed to the distribution maps of already quantitative magnetic characteristics obtained by the indirect method.

To probe the magnetic signal inside the cultivated cells, a micro-BLS setup was equipped with a high-resolution Sandercock (3 + 3)- tandem Fabry–Perot interferometer (TFPI), and a probe with a confocal microscope with the use of large numerical aperture (NA = 0.95) objectives and narrow spatial filters in order to have a resolution in the spin–wave wavelength was used. The distance between the mirrors in each of the two tandems was 6 mm in order to have a frequency resolution. To realize 2D mapping, the light with a wavelength of 532 nm and a power of 1 mW generated by single-frequency laser Excelsior (Spectra Physics, Andover, MA, USA) EXLSR-532-200-CDRH was focused on the cells inside the Petri dishes. It should be noted that the increase in laser power can be detrimental to live cells and tissues, and thus the method of extended acquisition time together with working at low laser power was used. Next, Petri dishes were placed in the in-plane magnetic field of value 3.2 kOe. The scattered light in the backscattering geometry was collected using the objective and then transmitted to the TFPI. The sensitivity of the BLS technique to the magnetic properties of the magnetite nanoparticles was previously obtained in [53] as a function of the external magnetic field value. It was demonstrated that the spectra of the in elastically scattered light the peak with a central frequency of about 12 GHz and a full width at half maximum of about 3 GHz is associated with light scattering on the magnons thermally excited in the magnetite particles. Inelastic light scattering on the thermally excited fluctuation of magnetic moments was confirmed by the shift in the central frequency under variation of magnetic field strength. To perform 2D mapping of the surface with the cells and magnetite nanoparticles, scanning 30 × 30 µm^2^ was performed using a Physik Instrumente Controller E-727.3CDA along with XYZ Piezo stage P-517.3CD. This method was used to plot a 2D color-coded map of the integrated BLS signal in the frequency domain. Next, to have the integrated BLS signal, summation over the whole scanning area and frequencies of the peak was performed. This approach serves as a tool for the comparison of the integrated BLS signal for the samples with varied numbers of submicron carriers per cell.

## 3. Results

### 3.1. Protein–Tannin–Mitoxantrone-Loaded Magnetic Carrier Formulation

**Figure 1:**
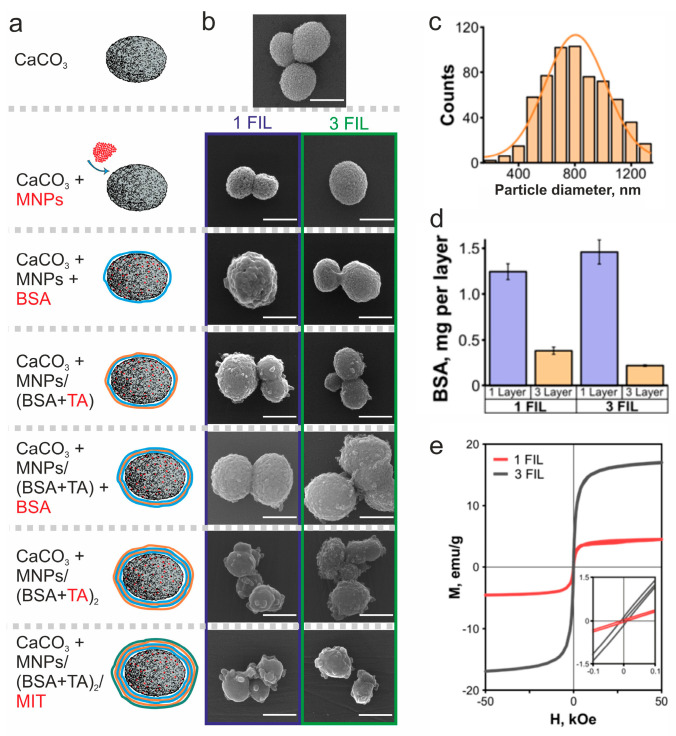
(**a**) Scheme of the step–by–step preparation of loaded magnetic carriers with protein–tannin–Mit shells; (**b**) SEM images demonstrating the change in morphology of the particles through the stepwise preparation of magnetic carriers with protein–tannin–Mit shells (scale bar—1 µm); (**c**) size distribution of initial CaCO_3_ particles; (**d**) dependence of BSA content in the carrier shell ofon the number of adsorption steps; (**e**) magnetization curves of CaCO_3_/MNPs particles with different MNP content at 300K.

Figure 1a,b show the SEM images and a schematic step-by-step representation of the process of preparation of self-degradable calcium carbonate magnetic carriers with biocompatible and biodegradable shells based on bovine serum albumin and tannic acid with the anticancer drug—mitoxantrone.

The particles have a porous structure and elliptic shape, which is typical for vaterite crystallites of submicron size. According to SEM, the average particle size is 950 ± 20 nm (Figure 1c) [54].

After MNP immobilization into vaterite particles by applying the FIL method, magnetic mineral CaCO_3_/MNPs composites with different contents of MNPs were obtained (Figure 1a,b). It should be noted that after one cycle of freezing and thawing of magnetic nanoparticles inside calcium carbonate pores, the surface of carriers looks more friable than after three such cycles, which correlates with the data obtained earlier [30]. Magnetic nanoparticles, by clogging the pores of calcium carbonate, smooth the surface of the particles. The amount of MPNs in composites after one FIL cycle was 1.4 ± 0.1 mg per 10 mg of CaCO_3_ and 7.1 ± 0.3 mg after three FIL cycles, which was calculated spectrophotometrically according to the method described in [30]. The various magnetic fraction content in CaCO_3_/MNP particles was confirmed by measuring their magnetization curves (Figure 1e). The saturation magnetization (M_s_) reaches 4.6 emu/g for CaCO_3_/MNP_1_ particles and 17.3 emu/g for CaCO_3_/MNP_3_particles respectively. Considering that the saturation magnetization of pure Fe_3_O_4_ nanoparticles is 75 emu/g, this corresponds to 6 and 23 wt% of Fe_3_O_4_ in the composite particles [55]. Furthermore, the measured M_s_ values of freezing-induced loaded submicron vaterite particles are four times higher than micron-sized vaterite particles loaded with magnetite by the co-precipitation technique [56]. Additionally, the CaCO_3_/MNP_1(3)_ particles demonstrated almost zero remanent magnetization, which is also inherent to superparamagnetic Fe_3_O_4_ nanoparticles. Furthermore, the absence of the remaining magnetization allows us to conclude that the freezing-induced loading of MNPs did not lead to the formation of nanoparticle clusters that behave as separate magnetic domains in the CaCO_3_ core. Therefore, the oscillation of magnetic moments in a single particle does not affect the oscillation of magnetic moments in neighbor particles, and the whole CaCO_3_/MNP core responds to reversal magnetization as a superparamagnetic particle. This suggests that the CaCO_3_/MNP_1(3)_ is capable of moving following the oscillation of the ELF AMF, which is an important prerequisite to magneto-mechanical actuation.

The protein–tannin shell on CaCO_3_/MNP_1_ and CaCO_3_/MNP_3_ composite particles was formed by the deposition of two TRITC-BSA/TA bilayers via layer-by-layer (LbL) assembly [14]. The adsorption of each protein layer is accompanied by smoothing the particle surface, which indirectly indicates protein adsorption. Bovine serum albumin is a large molecule, so the change in the surface structure can be detected with SEM. Tannic acid is a phenol and thus has a fundamentally different molecular structure, as well as a small molecular weight, when dissolved in water, which lowers the acidity to about three. In the acidic environment, which occurs upon the adsorption of tannic acid on the surface of calcium carbonate particles, the protein on the surface of the carriers partially changes its conformation, forming molecular tangles, which can be seen in the SEM images (Figure 1a,b). This observation also indirectly indicates the successful transfer of tannic acid during the adsorption process. This resulted in core–shell structures with various content of MNPs. The adsorption of TRITC-BSA was evaluated at every bilayer assembly. The resulting protein content was found to be 1.6 ± 0.1 mg for CaCO_3_/MNP_1_/(BSA-TRITC/TA)_2_ structures and 1.7 ± 0.1 mg for CaCO_3_/MNP_3_/(BSA-TRITC/TA)_2_structures (Figure 1d). In particular, most of the protein is adsorbed onto the CaCO_3_/MNP_1(3)_ surface after the assembly of the first bilayer. The non-proportional behavior of protein adsorption from the solutions of various concentrations onto the surface of calcium carbonate microparticles was shown earlier [57]. CaCO_3_/MNP_1(3)_/(BSA-TRITC/TA)_2_ can be attributed to the fact that protein adsorption takes place on different types of surfaces during the assembly of the first and the second bilayer. At first, the protein adsorbs onto the bare CaCO_3_/MNP_1(3)_ particle surface. In this case, the deposition is more likely to be governed by the physical adsorption onto the extended particle surface. In turn, the adsorption of the second protein layer takes place on the surface that has been already modified with the BSA/TA bilayer. After the assembly of the first bilayer, the surface becomes smoother, and, therefore, the efficiency of protein adsorption is more governed by its interaction and formation of hydrogen bonds with the TA layer. In this work, the concentration of TA was chosen experimentally in a way that the resulting core–shells should demonstrate a minimal aggregation. This resulted in the formation of a TA layer that can absorb only a limited amount of BSA, and, thus, reduces the efficiency of BSA adsorption compared to the first bilayer assembly.

Finally, CaCO_3_/MNP_1(3)_/(BSA-TRITC/TA)_2_core–shells were incubated in an Mit aqueous solution to prepare Mit-loaded magnetic drug carriers. According to the spectrophotometric measurements, the incubation led to the loading efficiency of Mitat about 99%, which means that almost all drugs were successfully loaded to the core–shells. In particular, the Mit loading was 198.7 ± 0.8 μg for CaCO_3_/MNP_1_/(BSA-TRITC/TA)_2_ core–shells and 197.2 ± 0.8 μg for CaCO_3_/MNP_3_/(BSA-TRITC/TA)_2_ core–shells, which corresponds to a concentration of adsorbed Mit to the mass of calcium carbonate particles of 19.9 ± 0.1 μg/mg for CaCO_3_/MNP_1_/(BSA-TRITC/TA)_2_ core–shells and 19.7 ± 0.1 μg/mg for CaCO_3_/MNP_3_/(BSA-TRITC/TA)_2_ core–shells. Such a high loading efficiency of Mit is explained by the ability of mitoxantrone to irreversibly bind to albumins by electrostatic interactions [58,59]. Figure 1b shows the SEM image of the resulting Mit-loaded magnetic drug carriers. For one and three FIL of MNPs, the change in the shape of calcium carbonate particles occurs as a result of the freezing-induced loading of the magnetite nanoparticles and the formation of the protein–tannin–Mit shells on their surface [30,59]. Since both of the core–shells demonstrated the same high loading of Mit, the carriers with a higher content of magnetic nanoparticles were chosen for further experiments of the exposure to ELF AMFs.

### 3.2. Safety of Model Submicron Carriers with Mitoxantrone under Non-Heating ELF AMFs

**Figure 2:**
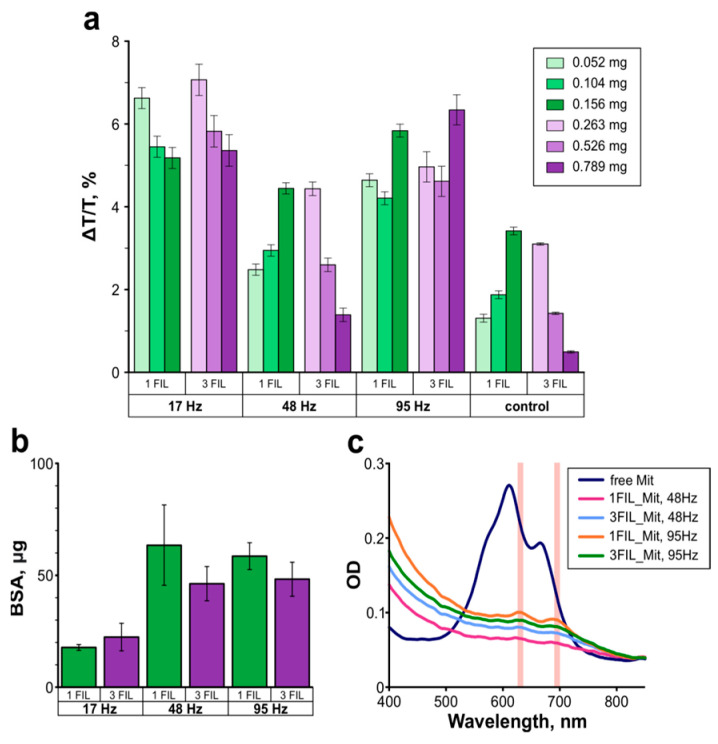
(**a**) Dependence of the relative temperature changes in samples in the ELF AMF treatment with 17, 48, and 95 Hz, and the MNPs content in the carriers (in mg) per well (temperature measurements were performed in a 96-well cell); (**b**) BSA release from protein–tannin magnetic mineral carriers after ELF AMF treatment with 0.156 mg and 0.789 mg magnetite content; (**c**) absorbance spectra of free mitoxantrone (0.012 mg in 0.1 M PBS) and Mit content supernatants from Mit-loaded protein–tannin magnetic mineral carriers with 0.156 mg and 0.789 mg magnetite content after ELF AMF treatment.

In the first set of experiments on the exposure of magnetic drug carriers to ELF AMFs, the absence of heating of carrier suspensions of various concentrations under the applied AMF was ensured. The initial suspensions of protein–tannin magnetic carriers were diluted with water by a factor of 2 and 3, relative to the content of magnetic nanoparticles (Table 1). To perform this, the suspensions of CaCO_3_/MNP_1(3)_/(BSA-TRITC/TA)_2_core–shells in a PBS buffer were prepared and exposed to ELF AMFs in a 96-well plate. The corresponding content of MNPs, CaCO_3_, and BSA per well in the resulting suspensions is given in Table 1. The exposure was performed at 17, 48, and 96 Hz frequencies with the magnetic field induction of 100 mT in a pulse mode. After exposure to AMFs, the thermographic analysis did not reveal the substantial heating of the liquid medium (Figure 2a). The relative growth of the temperature did not exceed 7.5%, regardless of the MNP content in the samples (Figure 2a). Furthermore, the control samples that were not exposed to AMFs demonstrated a 0.5% to 3.5% temperature increase, which indicates a 3% fluctuation in the environment temperature during the experiment (Figure 2a). Thus, the overall growth of the temperature of the exposed samples was only about 4% (Figure 2a). This confirms the non-heating nature of the employed AMF.

Further, the BSA release in the exposed suspensions was evaluated depending on the applied AMF frequency (Table 1). At an AMF frequency of 17 Hz, both of the core–shell samples demonstrated the same TRITC-BSA release of about 1 wt%, regardless of the BSA concentration. In turn, the increase in the AMF frequency to 48Hz was attended by the growth in BSA release up to 4 and 3 wt% for CaCO_3_/MNP_1_/(BSA-TRITC/TA)_2_ and CaCO_3_/MNP_1(3)_/(BSA-TRITC/TA)_2_ core–shells, respectively. The same was found at 96 Hz as well. All in all, the core–shells demonstrated a negligible release of protein upon exposure to the ELF AMF. A bit higher average BSA release from the core–shells assembled on the CaCO_3_/MNP_1_ cores prepared with one loading cycle of MNPs may be related to the lower overall mass of the particles, which makes them more responsive to magneto-mechanical actuation.

Finally, the release of Mit loaded to the BSA/TA shell under the ELF AMF was studied. The experiment was carried out at 48 and 96 Hz AMF with the lowest dilution of carrier suspension. Figure 2c shows the absorbance spectrum of the reference Mit solution in 0.1M PBS that has inherent maximums of absorbance at 610 and 665 nm. The collected upon exposure to AMF supernatants demonstrated a bathochromic shift of characteristic peaks to approximately 625 and 685 nm and very low optical density values (Figure 2c). This indicates the absence of Mit release owed to its irreversible binding to the BSA/TA shell and the formation of a strong protein–tannin–Mit complex [60], which successfully confirms obtaining a model carrier for further study on cell lines in magnetic fields.

Because vaterite with immobilized MNPs is a soft ferromagnetic material (see Figure 1e), the external magnetic field can lead to the movement of magnetic particles inside the vaterite matrix. The protein–tannin or protein–tannin–Mit shells were applied onto magnetic mineral particles, and the influence of immobilized MNP movement on the shell integrity under the ELF AMF was studied. For this purpose, samples with a higher magnetite concentration of 0.156 ± 0.004 mg and 0.789 ± 0.005 mg (MNP_1_ and MNP_3_, respectively) were used.

The effect of the ELF AMF treatment on the CaCO_3_/MNPs1/(BSA/TA)_2_ and CaCO_3_/MNPs_3_/(BSA/TA)_2_ carriers was determined spectrophotometrically by BSA measurement in the supernatant after treatment (Figure 2b). As is shown in Figure 2b, the BSA content in magnetic carriers was 1.6 ± 0.1 and 1.7 ± 0.1 mg for one and three FIL, respectively. After treatment at 17 Hz, the BSA amount is negligible, and carriers prepared with one and three FIL cycles of MNPs demonstrated the same BSA release, about 1%. After the treatment at 48 and 95 Hz, the amount of released BSA was two to three times higher. Moreover, the BSA release from carriers prepared with one FIL cycle was more intensive and reached almost 65 μg (4%).

Since the protein release from the protein–tannin shell was negligible at 17 Hz, the treatment at 48 and 95 Hz was chosen for further experiments in situ and in vitro. To confirm the obtained results of protein release from magnetic carriers, Mit-loading carriers with the highest magnetite content for one and three FIL were also subjected to ELF AMF treatment. Free mitoxantrone solution in 0.1M PBS has absorbance peaks of 610 and 665 nm (Figure 2c). Absorbance spectra of the supernatant after treatment are presented in Figure 2c and demonstrated a bathochromic shift of characteristic peaks to approximately 625 and 685 nm and very low optical density values. This indicates that Mit under the ELF AMF was not released from the protein–tannin shell but irreversibly binds to it due to the formation of a strong [BSA/TA/Mit] complex [60]. As was shown earlier, the cytotoxicity of mitoxantrone in the protein–tannin complex is high even at a dosage of 5 μg/mL [30]. Therefore, the amount of Mit released after ELF AMF treatment at 48 and 95 Hz will be insufficient to reduce cell viability.

Thermometry and spectrophotometry have shown that the ELF AMF treatment has no significant effect on mineral carriers with magnetic nanoparticles, covering the protein–tannin shell. Magnetic carriers with protein–tannin–Mit shells under ELF AMFs with different frequencies do not heat up, and a light release of protein and a non-release of Mit from the shell takes place. These results make protein–tannin–Mit shell magnetic carriers possible to further use obtained model magnetic systems in vitro on healthy and cancer cell lines without temperature effects.

We used the release of protein from drug submicron carriers under the action of the ELF AMF as a model system. Non-intense protein release (the studies were carried out using a spectrophotometer with a detection limit of 3 pM) indicates that the platform we have chosen for drug delivery is unique.

### 3.3. Internalization and Label-Free Visualization of Magnetic Core–Shell Structures into 4T1 Cells In Vitro

**Figure 3:**
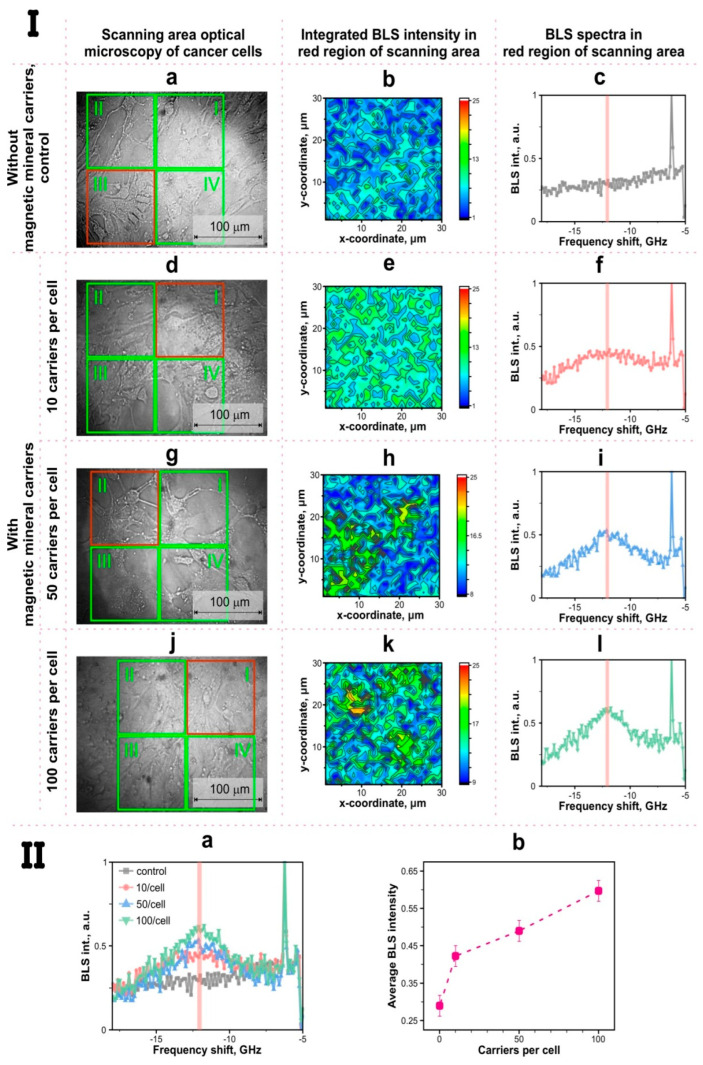
BLS 1. (**I**)—optical bright-field images of cancer 4T1 cells without (**a**) and with (**d**,**g**,**j**) different amounts of CaCO_3_/MNPs_3_/(BSA/TA)_2_protein—tannin magnetic mineral carriers; BLS maps (**b**,**e**,**h**,**k**) in the “red region” of the scanning area; pseudo color scale corresponds to BLS intensity on the z-coordinate in the “red region” (pseudo color-coded integrated BLS intensity is used); BLS spectra (**c**,**f**,**i**,**l**) in the I + II + III + IV regions of the scanning area. (**II**)—BLS spectra (**a**) obtained from all regions of the scanning area; average integrated BLS intensity dependence (**b**) of the added carriers per cell.

To confirm the suggestion that the core–shells are capable of moving within the cells under ELF AMF treatment, we applied the BLS cell investigation method. Particles with different concentrations (10, 50, and 100 carriers/cells) were added to the Petri dishes with 4T1 cancer cells and incubated together for 24 h. During 24 h of joint incubation, the particles are uptaken by the cells and enter the intracellular space [61]. Petri dishes were placed in the BLS microscope with a constant in-plane oriented magnetic field to acquire the in elastically scattered light, as is described in Section 2.11. Next, 2D mapping was performed in the regions denoted by red squares in optical bright-field images in Figure 3, part I(a,d,g,j) using the piezostage. In each 30 × 30 µm^2^ scanning area, in elastically scattered light was analyzed in the interferometer, and the BLS spectra were obtained. Next, the integration inside the frequency range from 8 GHz to 16 GHz was performed. The integrated BLS intensity was color-coded and plotted in Figure 3, part I(b,e,h,k). It is seen that the average BLS signal increases with the increase in the value of carriers per cell. Figure 3, part I(c,f,i.l) demonstrates the BLS spectra, which are the result of integration over the scanning range for each frequency. All spectra are normalized to the intensity of the Rayleigh peak and then normalized to have the same range from about 0 to 1. The peak at 6 GHz is associated with the parasitic laser peak, which has no influence on the magnetic peak. All obtained spectra are plotted in Figure 3, part IIa, and the average BLS intensity is plotted as a function of carriers per cell in Figure 3, part IIb. It should be noted that the BLS signal has a tendency to saturate with the increase in carriers per cell.

Figure 3 shows optical images of cells with carriers, a 2D BLS map of cells with magnetic carriers, and an average BLS spectrum for each concentration of magnetic carriers per cell. The intensity increases of the BLS peaks with a concentration increase in magnetic carriers (Figure 3, part I—c,f,i,l, and part IIa) per cell indicates that the magnetic component in the cells is growing, and in addition, this is a direct confirmation that the cells have magnetic properties. At the same time, the increase in the magnetic component in cells occurs non-linearly, which is explained by the limit of the possibilities of internalization of carriers into cells (Figure 3, part IIb). A comparison of red areas of optical images of cell lines with carriers (Figure 3, part I—a,d,g,j) and 2D BLS maps shows that the maximum of the magnetic signal is observed precisely in areas of large cells or near cell nuclei (Figure 3, part I—b,e,h,k).

### 3.4. Response of Mit-Loaded Magnetic Carriers inside Cancer 4T1 Cells to Extremely Low frequency Alternating Magnetic Field Treatment

**Figure 4:**
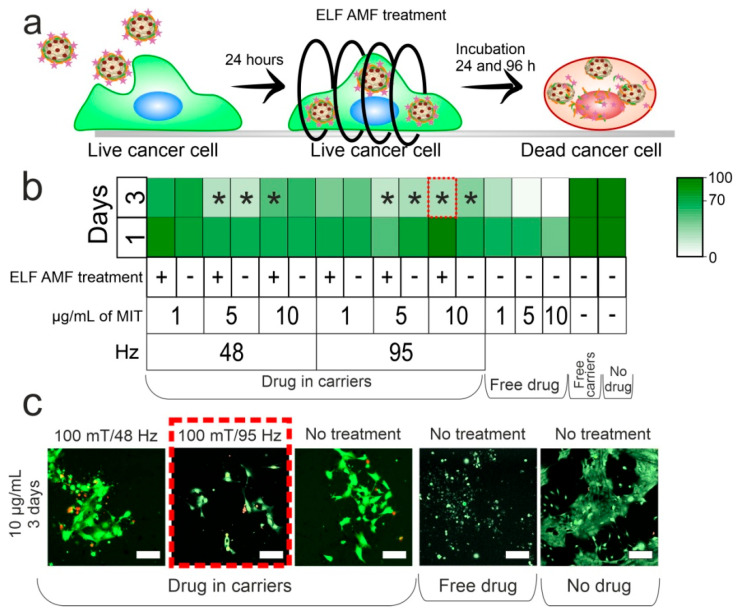
(**a**) Schematic representation of AF AMF treatment on the submicron carriers in the cells; (**b**) cell viability of 4T1 cancer cells under 48 and 95 Hz treatment by ELF AMFs (100 mT) at different concentrations of MIT (1, 5, 10 mg/mL) in the CaCO_3_/MNP_3_/(BSA/TA)_2_Mit carriers. An asterisk (*) indicates significant differences from the control cell group. The statistical analysis was performed by ANOVA followed by the Turkey test (*p* < 0.05). (**c**) CLSM—images of 4T1 cancer cells cultivated with carriers (10 μg/mL) during 3 days with loaded and free cytostatics after ELF AMF treatment. The scale bar is 30 μm. The red dashed line highlights images of cells that have the lowest cell viability.

The following set of experiments was devoted to the investigation of the effect of the ELF AMF on Mit-loaded magnetic carriers internalized by 4T1 cancer cells. The scheme of the experiment is shown in Figure 4a. We hypothesize that magneto-mechanical actuation of the drug carriers may have a double effect, which is a disruption of the cell membrane or interior along with the promotion of the drug release due to the mechanical motion of the carriers.

Mitoxantrone is a type II topoisomerase inhibitor of DNA synthesis and DNA repair in both healthy cells and cancer cells by the intercalation between DNA bases. In such a process, the decrease in cell viability is not spontaneous and takes time to induce apoptosis and necrosis [62].

A magnetic field strength of 100 mT with frequencies of 48 and 95 Hz was chosen. To compare the effect of a free cytostatic with an encapsulated one, it was convenient to operate with the concentration values expressed in μg/mL. In the experiment, we have analyzed two time points: 1 and 3 days (Figure 4b). After 1 day of joint incubation, we did not reveal any statistical difference between encapsulated and free cytostatic under ELF AMF treatment and without it. However, the MIT-loaded drug carriers containing 5 μg/mL of MIT after treatment with 95 Hz ELF AMFs and free MIT at a concentration of 10 μg/mL demonstrated the lowest average cell viability. These results are statistically significant and were reproduced with two different cell passages in triplicate.

After 3 days of co-incubation of cells with the carriers, we observed a decrease in the percentage of living cells for both exposure parameters. At 48 Hz and 1 μg/mL, cell viability was 82 and 88% for ELF AMF-treated cells and non-treated cells, respectively. The most successful use of this frequency can be considered the concentration of 5 μg/mL, at which the percentage of the surviving cells was 46 and 42%, which indicates the presence of a toxic effect on cancer cells [63]. However, there is no statistical difference between the viability of treated and untreated cells. For the 95 Hz ELF AMF treatment, the best result is also represented by a concentration of 5 μg/mL. The cell viability was 43.5 (red dashed line in Figure 4b) and 48.8% for ELF AMF-treated cells and non-treated cells, respectively. Compared to 5 μg/mL, the increase in MIT concentration to 10 μg/mL did not show a significant decrease in cell viability.

After that, we carried out a microscopic examination of the cells after incubation with the carriers. Photographs of cells incubated for 3 days with carriers containing MIT at a concentration of 5 μg/mL are shown in Figure 4c. As can be seen in the images, living cells have green fluorescence (calcein AM) and dead cells have red fluorescence (propidium iodide). For 48 Hz ELF AMF treatment, we can observe a large number of dead cells; however, living cells are also present in large numbers and look like normal adherent cells. For 95 Hz ELF AMF treatment (red dashed line in Figure 4c), the number of cells is much less and their size is much smaller, which may indicate a low cell adhesion and viability. Cells that were not exposed to the magnetic field but were incubated with particles outwardly resemble cells of 48 Hz ELF AMF treatment. This similarity suggests a significantly lower impact of 48 Hz treatment on the MIT release process in the cells. Cells incubated with the free cytostatic have a rounded morphology, which is explained by the practically absent adhesion, as well as the absence of red fluorescence, which indicates that the cells are alive, although they have a different morphology. The control cells practically formed large colonies in 3 days and practically became a monolayer, which indicates their good viability.

This result suggests that carriers penetrate into cells and release cytostatic molecules directly into their internal environment, which significantly accelerates the process of cell death. The free cytostatic does not kill cells as quickly (no red fluorescence) but significantly affects cell adhesion and proliferation. The ELF AMF treatment at 95 Hz/100 mT appeared better than the treatment at 48 Hz/100 mT, as the improved release of the cytostatic agent inside the cells successfully reduced their proliferation and viability. To conclude this section, we can assume that the effect of carriers on cell viability is not as significant as the effect of a free cytostatic; however, the experiments presented above in Section 3.2 show the release of substances loaded on the surface of carriers only in the presence of treatment. The experiments presented in Figure 4c show that the particles have excellent cellular uptake. As a consequence, we believe that only the carriers entering the cells will affect cell survival, and since the carriers are magnetic, it is possible to target the particles specifically to tumor cells. As a consequence, we believe that such a system can be extremely useful in reducing the number of active substances and improving local cancer therapy.

### 3.5. Release of Calcium and Iron Ions from Magnetic Cor–Shell Protein–Tannin Carriers in Enzyme Solutions and Buffers

**Figure 5:**
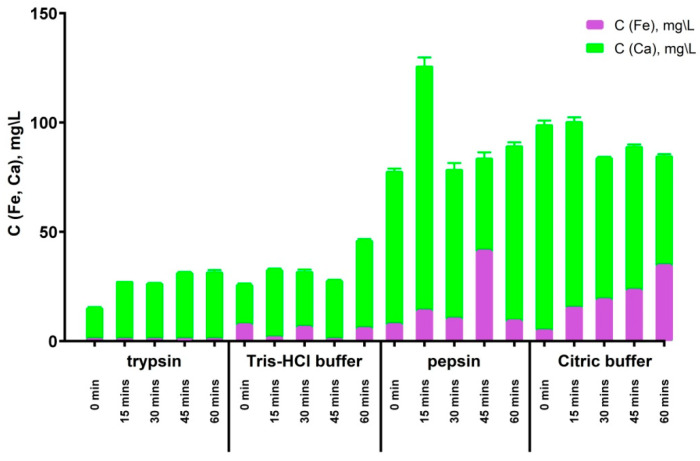
Concentrations of calcium and iron ions measured with AAS released from calcium carbonate-based magnetic carriers with protein–tannin shells in 1 mg/mL trypsin solution in 50 mM Tris-HCl buffer (pH 8.0) and 1 mg/mL pepsin solution in 50 mM citrate buffer (pH 4.0), and also in 50 mM Tris-HCl buffer (pH 8.0) and 50 mM citrate buffer (pH 4.0) during 0, 15, 30, 45, and 60 min.

The evaluation of the release of calcium and iron ions from magnetic carriers with protein–tannin rims into model solutions is an important parameter for determining the biological safety of the proposed carriers. As previously shown [48], calcium carbonate-based magnetic nanocarriers change their crystalline form from vaterite to calcite in pepsin and citrate buffer solutions within the first 30 min. This corresponds to the maximum possible release of calcium ions, as confirmed by the AAS data (Figure 5). Fifteen minutes after placing the carriers in the pepsin solution, the maximum amount of calcium ions in the supernatant is observed for all observed samples, while a higher amount of calcium ions is observed for particles in the pepsin and citrate buffer solutions for all times of observation compared with the release of calcium ions in the trypsin and Tris-HCl buffer solutions. This is due to the fact that in acidic media more intense dissolution of calcium carbonate particles occurs than in neutral and alkaline media. The maximum amount of iron ions is observed in the supernatants of particles that were in the pepsin solution for 45 min, and in citrate buffer, there is an almost linear increase in the concentration of iron ions in the supernatants, indicating the release of iron nanoparticles from the carriers over time.

The release of iron ions from the carriers in trypsin solution occurs passively in weakly alkaline media (trypsin and Tris-HCl buffer solutions), as well as forдляcalcium ions, which indicates the stability of the carrier core in weakly alkaline media (Figure 5).

The assumption about the possibility of calcium leaching by carbonate carriers in weakly alkaline environments of cancer cells can be realized [57]. The ratio of calcium carbonate particles per cell as well as exposure time and the possibility of cellular self-regulation of acidity are important [64]. The AAS data show that the release of iron ions in cellular experiments can be disregarded since the main release of iron ions occurs when the nucleus is destroyed or transformed into calcite (the time of complete conversion from vaterite to calcite depends on the particle size and can be 1 day [65]). In addition, iron ions can initiate lipid oxidation processes of polyunsaturated fatty acids in cells by the Fenton reaction. Therefore, the low level of free iron ions in weakly alkaline media for 1 h, at least, makes carriers safe. The presence of tannic acid in the carrier shells, which can act as a chelator and capture iron ions from solutions with physiological concentrations of iron ions and 10 times higher than that, thus providing protection against lipid oxidation, also allows us to significantly increase the safety of the carriers used for further experiments [66].

## 4. Conclusions

The effect of ELF AMFs on the release of a model protein (bovine serum albumin) from the shells of mineral magnetic submicron carriers was determined for three magnetic field frequencies of 17, 48, and 95 Hz at 100 mT in the pulsed mode. The thermometry of magnetic carriers in the ELF AMF shows that there is no significant heating of carriers in a pulsed magnetic field at fields of 17, 48, and 95 Hz at 100 mT. The release of the anticancer agent mitoxantrone from the carriers does not occur when using the same modes of action of the magnetic field, which indicates, together with the previously obtained results, that the mineral core–shell–mitoxantrone matrix formed by us is a depot system for the release of carriers. Since on the one hand, it contains an active surface for encapsulating drugs, on the other hand, it is not exposed to the action of a magnetic field when using a non-heating magnetic field, it decomposes under the action of proteolytic hydrolases [67] and has the potential to be used as an agent for theranostics, for example, in MRI [68]. The ability to bind to a protein, for example, anticancer drugs, will allow the implementation of a depot model of drug release under the action of the ELF AMF (in the case of mitoxantrone) or a model with an intensive release of the drug under the action of the ELF AMF (in the case of doxorubicin) [30]. The direct labelless BLS method, which was first applied to cell magnetic drug carrier systems, showed that the cells contain magnetic submicron carriers produced from 2D mapping. With regard to the suppression of the proliferation and survival of cancer cells, it was determined that the most optimal magnetic field parameters are 95 Hz and 100 mT. The use of the ELF AMF together with submicron mineral carriers of the anticancer substance effectively inhibits the activity of cancer cells in vitro. The encapsulated cytostatic shows a delay in reduced cell survival depending on the ELF AMF parameters from days 1 to 3. Our study was the first to calculate, in detail, the amount of calcium and iron ions that are released by enzymes over time, which can serve as a basis for further pharmacokinetic and pharmacodynamic studies.

We would like to emphasize that the novelty of this work lies in a number of new physicochemical data, which were obtained for the first time, namely, the behavior of magnetic carriers of drugs with cytostatics in the field of action of a non-heating alternating magnetic field and under the action of enzymes, the time-dependent effect of the concentration of magnetic carriers and magnetic field parameters on mouse breast cancer cells in vitro, the heating profile of magnetic carriers in the field of action of a non-heating magnetic field, and the mapping and magnetic characteristics of magnetic carriers in cells in vitro using the label-free BLS microscopy technique.

We believe that our step-by-step study has the potential to provide more detailed insight into the effects of ELF AMFs on cells in vitro with/without cytotoxic agents for use in biomedical research.

## Figures and Tables

**Table 1:** Evaluation of the content of protein, magnetic nanoparticles, calcium carbonate at one and three FIL of MNPs, and the BSA mass released under the action of a magnetic field (100 mT) of low frequencies. The highest values of released BSA under alternating magnetic fields treatment with frequencies of 17.48 and 96 Hz for samples with one and three cycles of the freezing/thawing of magnetic nanoparticles are highlighted in bold.

Number of FIL Cycles	MNP Content, mg	CaCO_3_Content, mg	BSA Content, Initial, mg	BSA Release, μg		
				17 Hz	48 Hz	95 Hz
One FIL	0.052 ± 0.004	0.4 ± 0.1	0.5 ± 0.1	8.3 ± 0.4	14.8 ± 5.2	22.6 ± 6.1
	0.104 ± 0.003	0.7 ± 0.1	1.0 ± 0.1	15.8 ± 5.3	15.8 ± 2.2	16.8 ± 2.5
	0.156 ± 0.004	1.1 ± 0.1	1.6 ± 0.1	**17.8 ± 1.3**	**63.5 ± 9.2**	**58.6 ± 6.0**
Three FIL	0.263 ± 0.005	0.4 ± 0.1	0.6 ± 0.1	6.4 ± 1.3	9 ± 3.9	9 ± 4.2
	0.526 ± 0.004	0.7 ± 0.1	1.2 ± 0.1	10.1 ± 3.8	14.5 ± 5.5	7.2 ± 2.6
	0.789 ± 0.005	1.1 ± 0.1	1.7 ± 0.1	**22.4 ± 6.2**	**46.3 ± 7.7**	**48.3 ± 7.6**

## Data Availability

All data are available online.
